# Disturbances in the FGFR1-5-HT1A Heteroreceptor Complexes in the Raphe-Hippocampal 5-HT System Develop in a Genetic Rat Model of Depression

**DOI:** 10.3389/fncel.2017.00309

**Published:** 2017-10-10

**Authors:** Dasiel O. Borroto-Escuela, Caitlin M. DuPont, Xiang Li, David Savelli, Davide Lattanzi, Ipsit Srivastava, Manuel Narváez, Michael Di Palma, Elisa Barbieri, Yuniesky Andrade-Talavera, Riccardo Cuppini, Yuji Odagaki, Miklos Palkovits, Patrizia Ambrogini, Maria Lindskog, Kjell Fuxe

**Affiliations:** ^1^Division of Cellular and Molecular Neurochemistry, Department of Neuroscience, Karolinska Institutet, Stockholm, Sweden; ^2^Department of Biomolecular Sciences, University of Urbino Carlo Bo, Urbino, Italy; ^3^Grupo Bohío-Estudio, Observatorio Cubano de Neurociencias, Yaguajay, Cuba; ^4^Instituto de Investigación Biomédica de Málaga, Facultad de Medicina, Universidad de Málaga, Málaga, Spain; ^5^Neuronal Oscillations Laboratory, Department of Neurobiology, Care Sciences and Society, Center for Alzheimer Research, Karolinska Institutet, Stockholm, Sweden; ^6^Department of Psychiatry, Faculty of Medicine, Saitama Medical University, Saitama, Japan; ^7^Department of Anatomy, Histology and Embryology, Semmelweis University, Budapest, Hungary

**Keywords:** fibroblast growth factor receptor 1, 5-HT1A receptor, heteroreceptor complexes, depression, hippocampus, raphe, dimerization, receptor-receptor interaction

## Abstract

The FGFR1-5-HT1A heteroreceptor complexes are involved in neuroplasticity in the rat hippocampus and in the mesencephalic raphe 5-HT nerve cells. There exists a 5-HT1A protomer enhancement of FGFR1 protomer signaling. Acute and 10 day treatment with intracerebroventricular (i.c.v.) FGF-2 and the 5-HT1A agonist 8-OH-DPAT produced enhanced antidepressant effects in the forced swim test (FST). We studied in the current work the disturbances in the FGFR1-5-HT1A heterocomplexes in a genetic rat model of depression, the Flinders sensitive line (FSL) rats of Sprague-Dawley (SD) origin, by means of neurochemical, neurophysiological and behavioral techniques. In control SD rats, the FGFR1 agonist SUN11602 and FGF2 produced a significant reduction of G protein-coupled inwardly rectifying K^+^ channel (GIRK) currents induced by 8-OH-DPAT in the CA1 area of the hippocampus. In FSL rats, only i.c.v. 8-OH-DPAT alone treatment produced a significant reduction in the immobility time. The combined i.c.v. treatment (FGF2 + 8-OH-DPAT) in FSL rats did not cause a significant decrease in immobility time in the FST. However, in the SD rats this combined treatment produced a significant reduction. Furthermore, in the FSL rat a significant increase in the density of FGFR1-5-HT1A proximity ligation assay (PLA) positive clusters was only found after i.c.v. 8-OH-DPAT treatment alone in the CA2 and CA3 areas. In the SD rat a significant increase in the density of specific PLA clusters was only observed in the CA2 area of the i.c.v. combined treatment (FGF2 + 8-OH-DPAT) group. No treatment led to significant changes in the PLA clusters of the dorsal raphe in the FSL rat. However, significant changes in the density of specific PLA clusters were only found in the dorsal raphe of SD rats after combined treatment and treatment with 8-OH-DPAT alone. The results indicate that in FSL rats compared with SD rats alterations may develop in the ability of 8-OH-DPAT and combined FGFR1 and 5-HT1A agonist treatment to increase the density of FGFR1-5-HT1A heteroreceptor complexes of the dorsal raphe. It is proposed that such deficits in FSL rats may possibly reflect a failure of the combined agonist treatment to uncouple the 5-HT1A autoreceptors from the GIRK channels. This may contribute to the failure of producing antidepressant-like effects in the FSL rat by combined agonist treatment as seen in the SD rat. The antidepressant-like effects seen with the 5-HT1A agonist alone treatment in FSL but not in SD rats may instead involve significant increases in the FGFR1-5-HT1A complexes of the CA2 and CA3 areas of the hippocampus.

## Introduction

In recent studies (Borroto-Escuela et al., 2012, 2013a,b,c) evidence is given for the existence of FGFR1-5-HT1A heterocomplexes based on the use of the *in situ* proximity ligation assay (PLA). They are involved in neuroplasticity in the rat hippocampus *inter alia* through the 5-HT1A protomer enhancement of FGFR1 protomer signaling. We have found that acute and a 10 day intracerebroventricular (i.c.v.) treatment with FGF-2 and the 5-HT1A agonist 8-OHDPAT in the Sprague-Dawley (SD) rat can produce enhanced antidepressant effects in the forced swim test (FST) vs. 5-HT1A agonist treatment alone (Borroto-Escuela et al., 2012). Thus, this cotreatment may possibly result in more rapid and stronger antidepressant actions than found with SSRIs, provided the agonist regulation of these heteroreceptor complexes is not disturbed in depression.

Evidence was also presented for the existence of FGFR1-5-HT1A heterocomplexes in the mesencephalic raphe 5-HT nerve cells (Fuxe et al., 2014; Borroto-Escuela et al., 2015a,b). The raphe 5-HT1A autoreceptor when being part of the FGFR1-5-HT1A heteroreceptor complexes may have a beneficial role in depression by assisting in the recovery of 5-HT nerve cell trophism including 5-HT synthesis and storage (Fuxe et al., 2014; Borroto-Escuela et al., 2016b).

Hippocampal pyramidal neurons and dorsal raphe nerve cells express G-protein-coupled inwardly rectifying K^+^ (GIRK) channels, which allow a slow inhibitory modulation of the overall cell excitability (Luscher et al., 1997). GIRK channels in raphe 5-HT neurons are assumed to be the main effectors of 5-HT1A autoreceptors. 5-HT1A autoreceptor-coupled GIRK channels were pharmacologically characterized in the dorsal raphe 5-HT neurons (Montalbano et al., 2015). It was found that nonselective potassium channel blocker like Ba^2+^ fully block the channels, while the GIRK specific blocker tertiapin-Q counteracted the 5-HT1A autoreceptor-activated GIRK conductance with high potency but with a 16% total conductance remaining (Montalbano et al., 2015). Furthermore, postjunctional hippocampal 5-HT1A receptors when activated by 5-HT in CA1 pyramidal neurons induces hyperpolarization (Luscher et al., 1997). Keeping this into consideration, FGFR1-5-HT1A heterocomplexes may diminish the autoreceptor function of the 5-HT1A protomer by reducing its coupling to the GIRK channels through FGFR1 protomer activation. This allosteric receptor mechanism may also exist in the postjunctional FGFR1-5-HT1A heteroreceptor complexes located on hippocampal pyramidal neurons (Borroto-Escuela et al., 2012, 2015a,b; Fuxe et al., 2014).

In the current article it was found through an electrophysiological analysis of the CA1 pyramidal neurons that FGF2 and a FGFR1 agonist reduced the 5-HT1A receptor agonist induced opening of the GIRK channels. Based on these and previous findings (Borroto-Escuela et al., 2012) the hypothesis was tested if disturbances in the combined receptor agonist regulation of FGFR1-5-HT1A heteroreceptor complexes can take place at the behavioral and neurochemical levels in an animal model of depression.

The model used was a selectively bred rat model of depression, the Flinders sensitive line (FSL) rat, using the naive SD rat as a control. The FSL rat strain is generated from the SD strain and is a well validated model that displays behavioral alterations resembling several symptoms of depression, including behavioral despair and memory deficits (Gómez-Galán et al., 2013; Overstreet and Wegener, 2013; Magara et al., 2015). Altered exploration strategies and reactive coping style were observed in the FSL rat model of depression (Magara et al., 2015). Also biochemical and electrophysiological changes related to the gene expression of serotonergic and glutamatergic neurotransmission were observed in the FLS rats (Treccani et al., 2016; Du Jardin et al., 2017). The acute effects of i.c.v. treatment with 8-OH-DPAT and/or FGF2 were evaluated in the FST and on the 5-HT1A-FGFR1 heterocomplexes in the dorsal hippocampus and the dorsal raphe of these two strains with *in situ* PLA techniques.

## Materials and Methods

### Animals

All experiments were performed using male SD rats (Scanbur, Sweden) or male FSL rats (bred in-house). The rats were 3–4 months of age at the time of behavioral testing. The animals were group-housed under standard laboratory conditions (20–22°C, 50%–60% humidity); animals that underwent surgery were single-housed after surgery. For the behavioral testing, the rats were handled for a minimum of 6 days before testing to minimize stress effects. Each animal was used for one test only. All experiments at the Karolinska Institutet were approved by the Stockholm North Committee on Ethics of Animal Experimentation. The study in Italy was performed in accordance with the current Italian legislation (D.lgs 26/2014) on animal experimentation, which is in strict accordance with the European Council Directives on animal use in research (n. 2010/63/EU).

### Electrophysiological Analysis of GIRK Currents

The experiments were carried out on adult male SD rats. After anesthetization with isoflurane and killing by decapitation, brains were quickly removed and incubated in chilled oxygenated solution, containing in mM: 110 choline Cl^−^, 2.5 KCl, 1.3 NaH_2_PO_4_, 25 NaHCO_3_, 0.5 CaCl_2_, 7 MgCl_2_, 20 dextrose, 1.3 Na^+^ ascorbate, 0.6 Na^+^ pyruvate, 5.5 kynurenic acid (pH = 7.4; 320 mosM). Hippocampal transversal slices (400 μm thick) were obtained from each hemisphere by vibrating microtome (Campden Instruments, Lafayette, IN, USA) and were allowed to recover in oxygenated artificial cerebrospinal fluid (aCSF) containing in mM: 125 NaCl, 2.5 KCl, 1.3 NaH_2_PO_4_, 25 NaHCO_3_, 2 CaCl_2_, 1.3 MgCl_2_, 1.3 Na^+^ ascorbate, 0.6 Na^+^ pyruvate, 10 dextrose (pH = 7.4; 320 mosM). The slices were kept in this solution for 1 h at room temperature before electrophysiological recordings. Each slice was then transferred into a recording chamber, where it was continuously superfused throughout electrophysiological recordings with oxygenated aCSF at a rate of 3 ml/min.

Patch clamp technique in whole cell configuration was used. The experiments were performed under visual guidance by a Zeiss Axioskop microscope (Carl Zeiss International, Italy) equipped with an infrared videocamera connected to a monitor. Recordings were carried out using an Axopatch-200B amplifier (Axon Instruments, CA, USA) and WinWCP software for data acquisition and analyses (Strathclyde electrophysiology software, John Dempster, University of Strathclyde, Glasgow, UK). Recording pipettes were pulled from borosilicate glass capillaries (World Precision Instruments, Sarasota, FL, USA) using a vertical puller (model PP-830 Narishige, Japan) and had 3–5 MΩ tip resistance. The internal solution contained in mM: 126 potassium gluconate, 8 NaCl, 0.2 EGTA, 10 HEPES, 3 Mg_2_ATP, 0.3 GTP (pH = 7.2; 290 mosM). No correction was made for junction potential between internal and external solutions. Somata of neurons to be recorded were identified in CA1 pyramidal cell layer based on their typical shape. Resting membrane potential (RMP) and input resistance (IR) were determined as previously described (Ambrogini et al., 2004).

In order to investigate GIRK channel currents, we performed experiments in voltage clamp mode (Voltage holding = −70 mV) for evaluating the holding current and the IR, following bath-application of specific agonists of 5-HT1A (5 μM 8-OH-DPAT) or of FGFR1 (10 μM SUN11602 or 10 ng/ml FGF2) or together in a mixture.

### ICV Drug Treatment

For ICV drug delivery animals were implanted with a guide cannula (Plastics One, Roanoke, VA, USA). Rats were anesthetized with isoflurane through a breathing mask. For some FSL rats, pentobarbitate was given to achieve full anestesia. Guide cannulas were implanted at the following coordinates relative to bregma and the dura surface: mediolateral: −1.2 mm; anteroposterior: −1.0 mm; dorsoventral: −3.7 mm; at a 0° angle from the vertical axis in the coronal plane (Paxinos and Watson, 1998). Animals were allowed to recover 6–7 days after surgery before experimental testing. Drugs were delivered 24 h before the testing day of the FST (i.e., right after the training session) and then again 24 h before sacrifice and collection of the brain (i.e., right after the testing session; see Supplementary Figure S1). Drugs were dissolved in aCSF and injected via a guide cannula using a microsyringe pump (1 μl/hemisphere) at the following final concentrations: FGF2 50 ng, 8-OH-DPAT 200 nmoles.

### Forced Swim Test

Immobility in the FST is used to measure behavioral despair and has good predictive value for testing antidepressant effects (Porsolt et al., 1977). Each rat (*N* = 8–16 rats/group) was placed for 15 min in a vertical Plexiglas cylinder (height: 50 cm; diameter: 30 cm) containing 37 cm of water (25 ± 1°C). The drugs were administered immediately after the first session through i.c.v. injections to allow 24 h after drug delivery before testing the behavior in the FST. Twenty-four hours later, the rat was placed in the cylinder for a second session and filmed for 5 min. A second dose of the drugs was then administered and the rats were sacrificed 24 h later by perfusion with 4% paraformaldehyde in PBS (for *in situ* PLA analysis) or decapitation (for binding analysis; see Supplementary Figure S1). Immobility time was scored manually by an observer who was blinded with respect to the experimental conditions and is defined as the cessation of activity aside from the absolute minimum movement required to remain afloat. Immobility items were compared as averages and statistical tests were made using GraphPad Prism Software.

### *In Situ* Proximity Ligation Assay

*In situ* PLA was performed as described previously (Borroto-Escuela et al., 2013c, 2016a). Free-floating formalin fixed brain sections (*n* = 5 per group) from male SD rats and FSL rats were employed using the following primary antibodies: rabbit monoclonal anti-5HT1A (vtg544, VTG Biosciences) and mouse monoclonal anti-FGFR1 (Abcam). Control experiments employed only one primary antibody or cells transfected with cDNAs encoding only one type of receptor. The PLA signal was visualized and quantified by using a confocal microscope Leica TCS-SL (Leica, USA) and the Duolink Image Tool software.

### Serotonin 5-HT1A Radioligand Binding Assay

[^3^H]-8-OH-DPAT; 141 Ci/mmole) was obtained from Perkin Elmer (USA). Ipsapirone, a 5-HT1A agonist (Glaser and Traber, 1985), as well as other basic chemicals used in buffers preparation were obtained from Sigma Aldrich (Stockholm, Sweden). Frozen hippocampal rat tissue was homogenized with an Ultra-Turrax in 5 mL of ice-cold preparation buffer containing 50 mM Tris-HCl/Tris-base and 2.0 mM EDTA (pH 7.4). The membranes were precipitated by centrifugation at 20,000 rpm for 10 min, 4°C. The resulting pellet was re-suspended in the same volume of preparation buffer, preincubated at 37°C for 10 min and centrifuged at 20,000 rpm for 10 min, 4°C, three times. The final pellet was re-suspended in preparation buffer, sonicated for 10 s and the total protein concentration was determined. The saturation experiment of 5HT_1A_ receptor binding was performed using 96-well microplates with GF/B filter (Perkin Elmer, Waltham, MA, USA), using eight concentrations of [^3^H]-8-OH-DPAT (in the range of 0.116–20 nM). The assay mixture (total volume of 300 μl) contained the membrane suspension (80 μg of protein per reaction), and [^3^H]-8-OH-DPAT in the buffer containing 50 mM Tris-HCl, 10 μM pargyline, 10 mM MgCl_2_ 6H_2_O and 0.1% ascorbic acid. For nonspecific binding 10 μM ipsapirone was used. The reaction mixture was incubated at RT for 60 min with gentle shaking. The binding was terminated by rapid filtration, followed by three washes with 200 μl of cold washing buffer (50 mM Tris-HCl, pH 7.4). The filters were dried overnight, and immersed in 2 ml of scintillation liquid (Ultima Gold MV, Perkin Elmer, Waltham, MA, USA). The bound ligand was determined by WALLAC 1409 DSA liquid scintillation counter. The results were calculated in GraphPad Prism 6.0 (GraphPad Software Inc., USA) and expressed as fmol/mg of total protein for B_max_ and in nM for K_d_ values. All radioligand experiments at the Karolinska Institutet were approved and performed in accordance with the current Swedish legislation (Stralsäkerhetsmyndigheten, 20 stralskyddslagen 1988:220).

### Data Analysis

The number of samples (*n*) in each experimental condition is indicated in Figure legends. All data were analyzed using the commercial program GraphPad PRISM 6.0. When two experimental conditions were compared, statistical analysis was performed using an unpaired *t* test. Otherwise, statistical analysis was performed by one-way analysis of variance (ANOVA) followed by Tukey’s (neurochemistry) or Dunnett (behavioral analysis) Multiple Comparison post-test. The *P* value 0.05 and lower was considered significant. *P < 0.05, **P < 0.01, ***P < 0.001.

## Results

### Electrophysiological Analysis of 5-HT1A Activated Hippocampal GIRK Currents and their Modulation by FGF2 and SUN 11602 in SD Rats

**Figure 1:**
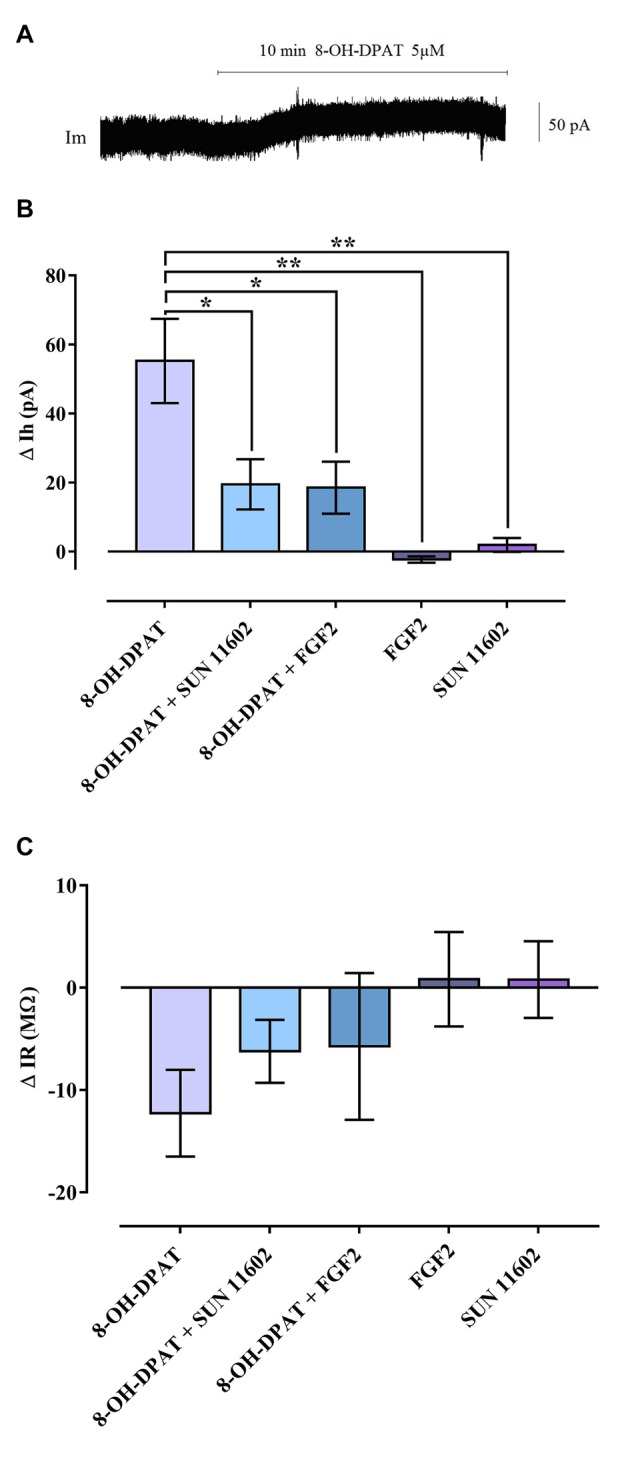
Reduction of G Protein-coupled inwardly rectifying K^+^ channel (GIRK) currents induced by combined bath application of 8-OH-DPAT and FGFR1 agonists on CA1 hippocampal neurons of Sprague-Dawley (SD) rats. **(A)** Holding current (Ih) trace of a CA1 pyramidal cell in voltage clamp, which shows the Ih shift following 5 μM 8-OH-DPAT application, indicating the occurrence of hyperpolarization. **(B)** Summary graph including all agonists tested. Combined application of 8-OH-DPAT together with FGFR1 agonists (10 ng/mL FGF2; 10 μM SUN 11602) significantly reduced the amplitude of the GIRK current induced by 5HT1A activation. One-way analysis of variance (ANOVA), Tukey’s *post hoc*: *p < 0.05; **p < 0.01. **(C)** GIRK channel opening decreased input resistance (∆IR = IR during drug application—IR baseline) of CA1 neurons. In line with the effect exerted on holding current, combined agonist treatment tended to reduce the IR drop elicited by 5HT1A-induced GIRK activation. All data are expressed as Mean ± SEM. Number of recorded cells (n): 8-OH-DPAT (8), 8-OH-DPAT + SUN 11602 (10), 8-OH-DPAT + FGF2 (8), FGF2 (6), SUN11602 (6). For further details see also Supplementary Figures S2–S4.

Activation of 5-HT1A induced an outward Gi/o mediated current in recorded CA1 hippocampal neurons due to GIRK channel opening (Figure 1) in line with the literature (Luscher et al., 1997). After application of 8-OH-DPAT (5 μM) in the bath perfusion, the 5-HT1A agonist produced a shift of holding current, demonstrating a hyperpolarization that increased over 10 min and was associated with a decrease of IR (Figure 1). In contrast, FGFR1 activation, by using the specific agonist SUN116052 (10 μM) or FGF2 (10 ng/ml), did not result in any effect on holding current in CA1 pyramidal neurons (Figure 1B), indicating failure of channel opening. Importantly, following co-application of 5 μM 8-OH-DPAT and 10 μM SUN11602, the outward current was greatly reduced in the CA1 pyramidal neurons (Figure 1). The same effect was also achieved by applying a mixture of 5 μM 8-OH-DPAT and FGF2 10 ng/ml (Figure 1). These data suggest that FGFR1 activation was able to produce a significant reduction of the GIRK current induced by 8-OH-DPAT. All observed effects were reversed by washing out with aCSF.

There was a non-significant trend for the FGFR1 agonist and FGF2 to counteract the reduction of IR produced by the 5-HT1A agonist (Figure 1).

### Effects of Acute i.c.v. Treatment with FGF2 and/or 8-OH-DPAT in the Forced Swim Test

#### SD Rats

**Figure 2:**
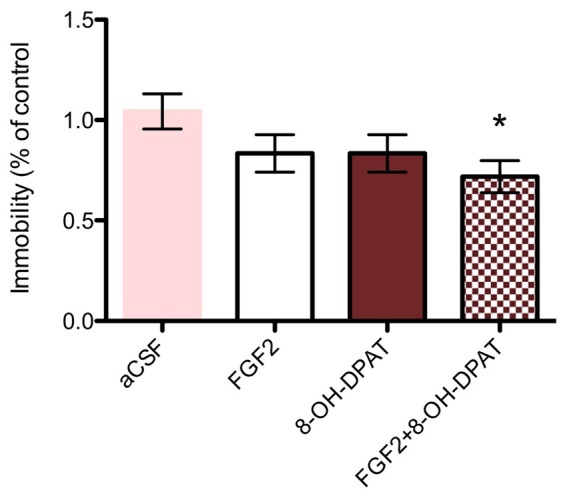
SD rats (SD, control strain) showed a significant reduction of immobility time upon combined 8-OH-DPAT and FGF2 intracerebroventricular (i.c.v) treatment in the forced swim test (FST) compared to vehicle controls. All drugs were administered i.c.v. 24 h before testing. Immobility time was scored during a 5 min test session by an experimenter blind to treatment conditions and expressed as percent of immobility time in vehicle treated controls. Mean ± SEM is shown. Neither FGF2 (50 ng) nor 8-OH-DPAT (200 nmoles) alone did significantly decrease immobility time in the SD rats. However, co-administration of the drugs showed a significant decrease in immobility time as compared to artificial cerebrospinal fluid (aCSF) controls (*p < 0.05, 1-way ANOVA). aCSF group *n* = 16 rats, 8-OH-DPAT group *n* = 11 rats, FGF2 group *n* = 9 rats, combined group *n* = 15 rats.

Each drug treatment by itself did not significantly affect the behavior in the FST (*n* = 9–11) compared to aCSF treated littermates (*n* = 16). However, the combined treatment of FGF2 (50 ng) and 8-OH-DPAT (200 nmoles) caused a decreased immobility time compared to aCSF treated littermates (Figure 2; *p < 0.05, 1-Way ANOVA followed by Dunnett multiple comparison test, *n* = 15–16 rats per group).

#### FSL Rats

**Figure 3:**
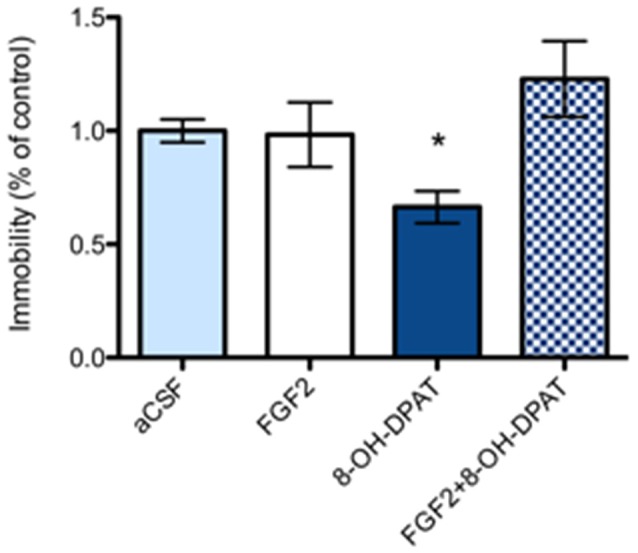
Flinder’s sensitive line (FSL) rats (“depressed” strain) showed a significant reduction of immobility time in 8-OH-DPAT alone (200 nmoles i.c.v.) treated rats in the FST compared to vehicle treated rats. All drugs were administered i.c.v. 24 h before testing. Immobility time was scored during a 5 min test session by an experimenter blind to treatment conditions and expressed as percent of immobility time in vehicle treated FSL rats. Mean ± SEM is shown. FSL rats demonstrated a significant decrease in immobility time with 8-OH-DPAT (200 nmoles) alone (*p < 0.05, 1-way ANOVA, *n* = 10–11), but not with FGF2 alone (50 ng, *n* = 8) nor with combined treatment) FGF2 + 8-OH-DPAT, *n* = 11). aCSF group *n* = 11 rats, 8-OH-DPAT group *n* = 10 rats, FGF2 group *n* = 8 rats, combined group *n* = 11 rats).

In FSL rats, a single i.c.v. treatment with 8-OH-DPAT alone (200 nmol/L) caused a significant reduction in the immobility time compared to aCSF treated littermates (Figure 3; *p = 0.05, 1-Way ANOVA followed by Dunnett multiple comparison test, *n* = 10–11 rats per group). In contrast, neither the combined treatment (*n* = 11), nor FGF2 by itself (*n* = 8) had any significant effect on immobility time in the FST.

### Effects of Acute i.c.v. Treatment with FGF2 and/or 8-OH-DPAT on Hippocampal FGFR1-5-HT1A Heterocomplexes Using the *in Situ* Proximity Ligation Assay

#### SD Rat

**Figure 4:**
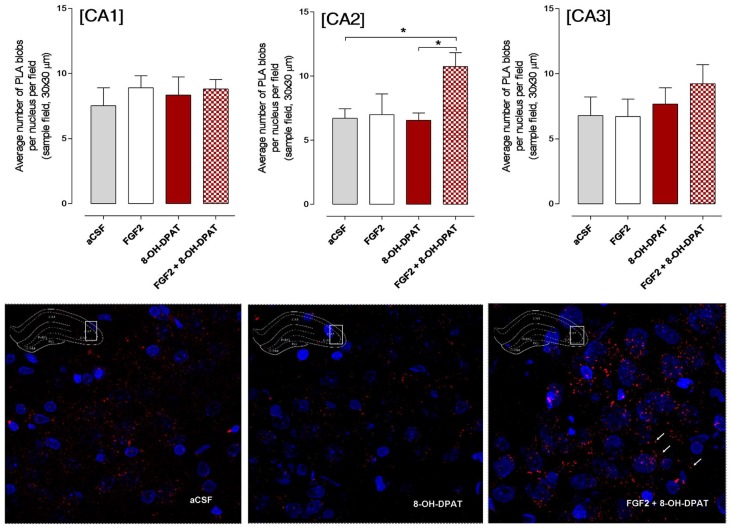
SD rats (controls) showed a significant increase in FGFR1-5HT1A heteroreceptor complexes (proximity ligation assay (PLA) positive clusters) in the CA2 area of the dorsal hippocampus following a combined but not single i.c.v. treatment of the two agonists, 24 h after drug administration. Drugs were administered i.c.v. immediately after the test session of the FST (FGF2 50 ng, 8-OH-DPAT 200 nmoles). All animals were euthanized 24 h later by a lethal dose of pentobarbital (200 mg/kg) and perfusion with formalin. PLA was quantified as PLA per nucleus per field by an experimenter blind to treatment conditions. FGFR1-5HT1A heteroreceptor complexes remain unchanged in SD rats in the CA1 subregion of dorsal hippocampus (no significance, Means ± SEM, five rats per group, quatriplicates, 1-way ANOVA). However, they increased significantly in the CA2 subregion upon combined but not single treatments vs. the vehicle and 8-OH-DPAT alone treated groups (Means ± SEM, five rats per group, duplicates, 1-Way ANOVA with the Bonferroni *post hoc* test *p < 0.05). The CA3 subregion did not show any significant changes to combined or single treatments but a non-significant increase was found with combined treatment (Means ± SEM, five rats per group, duplicates, 1-Way ANOVA). The panels below give representative examples of the significant increase of the density of PLA blobs in the CA2 area after the combined 8- OH-DPAT and FGF2 treatment compared with aCSF. The sampled field is centered on the pyramidal cell layer but includes also substantial parts of the stratum oriens and radiatum having reduced numbers of PLA positive clusters.

The CA1, CA2 and CA3 areas of the dorsal hippocampus were analyzed by confocal laser microscopy in fields mainly containing the pyramidal cell layer (Figure 4). The specific PLA clusters had a similar density in all these areas as seen from the number of PLA clusters per nucleus per sampled field. In Figure 4 it is seen that the only change found in the different treatment groups, in which the FST had been performed, was in the CA2 area. A significant increase in the density of FGFR1-5-HT1A PLA clusters was observed in this region in the combined treatment (FGF2 + 8-OH-DPAT) group. A possible trend for such an increase was found in the CA3 but not in the CA1 area in the combined treatment group (Figure 4). The panels below the quantitation graphs illustrate the increases obtained with the combined treatment in the CA2 area vs. 8-OH-DPAT alone and aCSF alone. The number of PLA positive cells in percent of total number of nuclei per sampled field did not change in any region (data not shown).

#### FSL Rat

**Figure 5:**
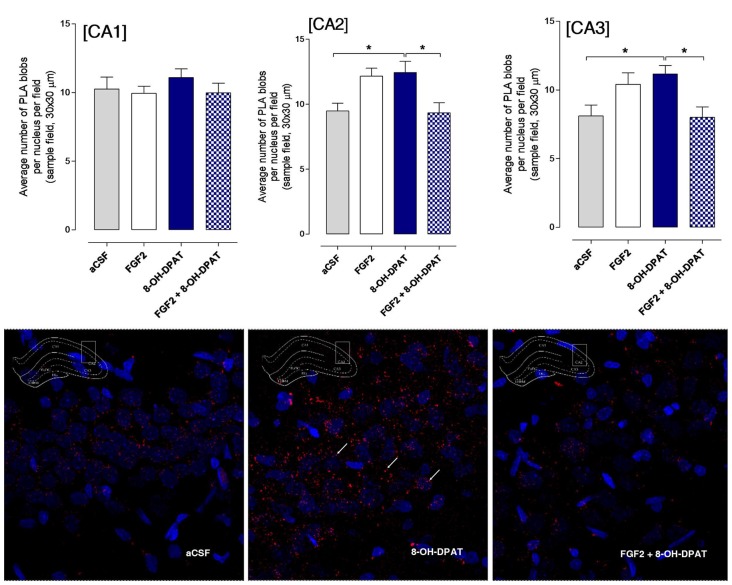
Hippocampal FGFR1-5-HT1A heteroreceptor complexes (PLA clusters) increase in the CA2 and CA3 areas of the dorsal hippocampus in FSL rats following i.c.v. injections of 8-OH-DPAT alone but not with combined treatment. Drugs were administered i.c.v. immediately after the test session of the FST (FGF2 50 ng, 8-OH-DPAT 200 nmoles alone or combined). All animals were euthanized 24 h later by a lethal dose of pentobarbital (200 mg/kg) and perfused with formalin. PLA positive clusters were quantified as PLA clusters per nucleus per field by an experimenter blind to the treatment conditions. FGFR1-5HT1A heteroreceptor complexes remain unchanged in FSL rats in the CA1 area after acute treatment (Means ± SEM, five rats per group, duplicates, 1-Way ANOVA). In the CA2 area there is a significant increase of PLA clusters after 8-OH-DPAT treatment alone which was blocked by combined treatment (Means ± SEM, five rats per group, duplicates, 1-way Way ANOVA with Bonferroni *post hoc* test *p < 0.05). Also in the CA3 area 8-OH-DPAT increased the FGFR1-5HT1A heteroreceptor complexes (PLA clusters) in FSL rats, an action again blocked by combined agonist treatment (Means ± SEM, five rats per group, duplicates, 1-way ANOVA followed by Bonferroni *post hoc* test, *p < 0.05). The number of PLA positive cells in per cent of total number of nuclei per sampled field did not change in any region. Thus, the increase induced by 8-OH-DPAT in the number of PLA blobs per nucleus per sampled field in CA2 and CA3 areas reflects an increase in the density of blobs in already PLA positive cells. The panels give representative examples of the significant increase of the density of PLA blobs in the CA2 area after the 8-OH-DPAT alone treatment compared with aCSF and combined treatment.

A significant increase in the density of specific PLA clusters was found after i.c.v. treatment with 8-OH-DPAT treatment alone in the CA2 and CA3 areas of the dorsal hippocampus vs. both the aCSF controls and combined treatment groups (Figure 5; *<0.05, 1-Way ANOVA, Tukey’s Multiple Comparison post-test). The increase induced by i.c.v. FGF2 treatment alone did not reach significance. No effects were found in the CA1–3 areas by combined i.c.v. treatment with FGF2 and 8-OH-DPAT. In the panels below the graphs of quantitation, the increases of PLA positive FGFR1-5-HT1A heterocomplexes found are illustrated in the CA2 area (Figure 5).

The number of PLA positive cells in per cent of total number of nuclei per sampled field did not change in any region. Thus, the increase induced by 8-OH-DPAT in the number of PLA clusters per nucleus per sampled field in CA2 and CA3 areas reflects an increase in the density of blobs in already PLA positive cells.

### Effects of Acute i.c.v. Treatment with FGF2 and/or 8-OH-DPAT on Dorsal Raphe FGFR1-5-HT1A Heteroreceptor Complexes Using the *in Situ* PLA

#### SD Rat

**Figure 6:**
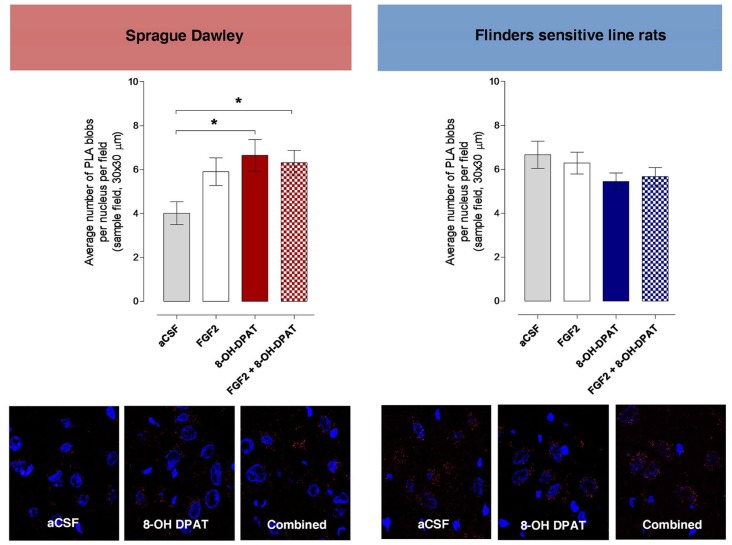
Dorsal raphe FGFR1-5-HT1A heteroreceptor complexes (PLA blobs/clusters per nucleus per sampled field) increase in the SD but not in the FSL rats following i.c.v. injections of 8-OH-DPAT and FGF2 alone or with combined treatment. Drugs were administered i.c.v. immediately after the test session of the FST (FGF2 50 ng, 8-OH-DPAT 200 nmoles alone or combined). All animals were euthanized 24 h later by a lethal dose of pentobarbital (200 mg/kg) and perfused with formalin. PLA positive clusters were quantified as PLA clusters per nucleus per field by an experimenter blind to the treatment conditions. FGFR1-5HT1A PLA clusters are increased in the dorsal raphe from SD control rats upon combined treatment and with 8-OH-DPAT treatment alone but remain unchanged in FSL rats after acute combined or single treatments. SD rats: Means ± SEM, 4–5 rats per group, duplicates, 1-Way ANOVA with Bonferroni *post hoc* test *p < 0.05); FSL rats: Means ± SEM, 4–5 rats per group, duplicates, 1-Way ANOVA, No significant differences. The panels give representative examples of the changes observed on the density of PLA blobs in the dorsal raphe area after the treatments.

The density of the specific PLA clusters in the dorsal raphe cells was found to be significantly increased to about the same degree by 8-OH-DPAT treatment alone and by combined FGF2 + 8-OH-DPAT treatment vs. the vehicle group (Figure 6; *<0.05, 1-Way ANOVA, Tukey’s Multiple Comparison post-test). The increase found with FGF2 alone did not reach significance. The results of the treatments on PLA cluster densities are illustrated in the panels below the quantitation graphs (Figure 6).

#### FSL Rat

No changes in the density of PLA clusters were observed after i.c.v. treatment with FGF2 and 8-OH-DPAT alone or in combination (Figure 6).

### ^3^H-8-OH-DPAT Binding Sites in the Hippocampus of the SD Control and FSL Rat Strains

**Figure 7:**
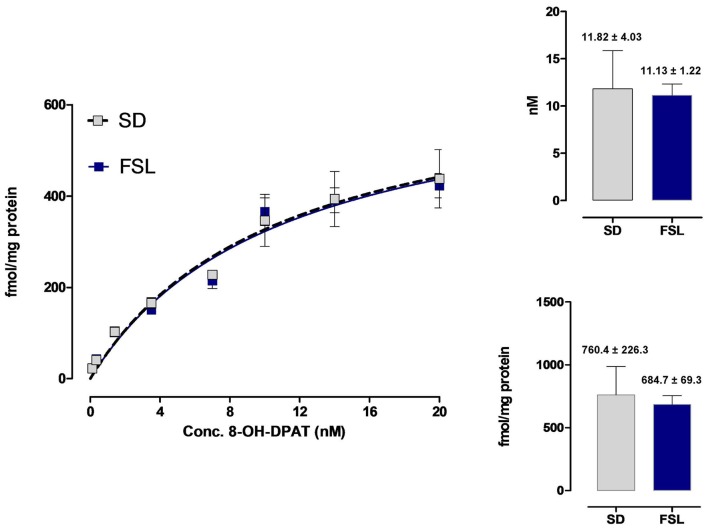
Saturation binding analysis of the 3H-8-OH-DPAT agonist binding sites in the hippocampus of SD and FSL rats. The eight concentrations of the 5-HT1A radioligand ranged from 0.1 nM to 20 nM. Five rats from each group were used. Almost identical saturation curves were obtained for the hippocampal 5-HT1A binding sites. The B_max_ values for SD and FSL rats (fmoles/mg protein) and the K_d_ values (nM) are indicated in the right panels.

This analysis was performed to test if any significant strain differences existed in the affinity and density of the 5-HT1A receptor agonist binding sites. As seen in Figure 7, the saturation binding analysis showed no significant difference in the B_max_ and K_d_ values of the hippocampal high affinity 5-HT1A agonist binding sites between the two strains. The mean B_max_ values were in the SD rats 760.4 ± 223.3 fmoles/mg protein (*n* = 5) and in FSL rats 684.7 ± 69.3 fmoles/mg protein (*n* = 5). The mean K_d_ values were 11.82 ± 4.03 nM (*n* = 5) in the SD rats and 11.13 ± 1.22 nM (*n* = 5) in the FSL rats.

## Discussion

The electrophysiological analysis of 5-HT1A activated GIRK currents in CA1 hippocampal neurons gave evidence that FGF2 and the FGFR1 agonist SUN11602 can substantially and significantly reduce the GIRK current by 8-OH-DPAT. Thus, an uncoupling of the 5-HT1A Gi/o mediated opening of the GIRK channels may take place upon agonist coactivation in hippocampal CA1 neurons of the SD rat of the FGFR1-5-HT1A protomers forming heteroreceptor complexes located on the CA1 pyramidal nerve cells. In line with these results it was previously found by electron microscopical studies that 5-HT1A are located on the plasma membrane of pyramidal hippocampal neurons at the soma and dendrite level (Riad et al., 2000). They were not present in synapses and therefore mainly mediating 5-HT volume transmission (Fuxe and Agnati, 1991). The 5-HT1A modulates the internal circuitries of the hippocampus upon activation by 5-HT released from widespread 5-HT nerve terminal networks (Steinbusch, 1981; Bjarkam et al., 2003) originating from the midbrain raphe region (Dahlstroem and Fuxe, 1964; Fuxe and Jonsson, 1974). It therefore became of interest to study the FGF2 and 5-HT1A agonist regulation of the FGFR1-5-HT1A heterocomplexes in the hippocampus and dorsal raphe in a genetic rat model of depression (FSL) vs. the control SD strain using the *in situ* PLA.

The existence of FGFR1-5-HT1A heterocomplexes in the hippocampus and the dorsal raphe (Borroto-Escuela et al., 2012, 2015a,b) could be validated in the SD rat in the current study. They could also be demonstrated in the FSL rat. In the FST it was again established that in SD rat combined but not single i.c.v. treatment with 8-OH-DPAT and FGF2 produced antidepressant-like effects as seen from the significant reduction of the immobility time observed (Borroto-Escuela et al., 2012). More importantly in the FSL rat there was no reduction in the immobility time in the FSL rat after combined i.c.v. treatment. It is of substantial interest that in the FSL rat no reduction of immobility time was observed after the combined treatment but only after i.c.v. treatment with 8-OH-DPAT alone. These results indicate that the FGF2 and 8-OH-DPAT interactions are disturbed in this genetic rat model of depression, which could involve alterations in the allosteric receptor-receptor interactions in the FGFR1-5-HT1A heterocomplexes. In fact, the density and affinity of the 5-HT1A agonist binding sites were not changed in the untreated FSL rat vs. the untreated SD rat as demonstrated in the current study. Nevertheless, 5-HT1A receptor supersensitivity has been demonstrated in the FSL rats vs. controls (Wallis et al., 1988; Shayit et al., 2003). Instead, alterations in the receptor-receptor interactions may be related to changes in the composition and stoichiometry of the FGFR1-5-HT1A heterocomplexes in the FSL vs. the SD strains. Also, it should be considered that there is an indication for a possible increase of the 5-HT1A-FGFR1 heteroreceptor complexes in the dorsal raphe of the FSL strain vs. the control strain. Such a change may block the ability of 5-HT1A receptor agonist and/or FGF2 treatment to produce a further increase of these heteroreceptor complexes in the FSL strain. It could also involve changes in the allosteric receptor-receptor interactions due to differences in the transmitter panorama between the two strains (Fuxe and Borroto-Escuela, 2016). These possibilities will be explored in further work including a neurophysiological analysis to test if there is a reduced 5-HT1A autoreceptor coupling to GIRK channels in the dorsal raphe of the FSL vs. the control strain due to an increase in the 5-HT1A-FGFR1 heteroreceptor complexes. Such an increase may be part of a compensatory mechanism to increase activity in the ascending 5-HT pathway from the dorsal raphe in this genetic model of depression.

We should also consider that in addition to the depression a stress factors could contribute to the obtained results since the recovery time after the surgery was only 6 to 7 days. Furthermore, the stress produced by the FST 24 h before sacrifice may possibly remain to a minor degree at the time of sacrifice.

The molecular mechanism may therefore involve differential changes in the receptor agonist regulation of the FGFR1-5-HT1A heterocomplexes through differential receptor-receptor interactions in the FSL vs. the SD rat. In the current study the brains of the FSL and SD control rats used in the FST were taken to *in situ* PLA 24 h after the FST with an additional i.c.v. treatment of FGF2 and 8-OH-DPAT alone or combined. In line with the behavioral results, the combined treatment but not the single treatments in the SD rat resulted in a significant and differential increase in the number of FGFR1-5-HT1A heterocomplexes in the pyramidal cell layer of the CA2 vs. the CA1 area with a trend for a similar change in the CA3 area. Such a recruitment of FGFR1-5-HT1A complexes in the CA2 area may contribute to the antidepressant-like effects observed by the combined i.c.v. treatment as previously found (Borroto-Escuela et al., 2012). The enhanced integrated response in the CA2 area through the increase in these heterocomplexes may be beneficial for the emotional brain circuits modulated by CA2 and participate in the antidepressant-like effects observed. Cell type-specific genetic and optogenetic tools demonstrated that hippocampal CA2 circuits involve synaptic inputs from the granular layer of the dentate gyrus and projections to the CA1 area (Kohara et al., 2014). Unlike the CA3 trisynaptic circuit, the CA2 trisynaptic circuit mainly targets the deep layer CA1 pyramidal cells which may have differential projections vs. the superficial CA1 cells (Kohara et al., 2014). They may also more effectively control the ventral CA1 projections to the basolateral amygdala and the prefrontal cortex (Cenquizca and Swanson, 2007). It was also recently demonstrated that the CA2 area is crucial for social memory based on a study involving a selective inactivation of CA2 pyramidal neurons through a genetic approach (Hitti and Siegelbaum, 2014). Again, a link of the CA2 area to emotional circuits receives support, underlining a putative role of the CA2 circuit in depression as indicated from the current findings.

In contrast, in the FSL rat the combined treatment failed to change the density of the PLA clusters in the CA2 and CA3 areas in line with the failure of the combined treatment in the FSL rat to produce antidepressant-like actions in the FST. It is of interest to see that 8-OH-DPAT alone instead produced a significant increase in the FGFR1-5-HT1A heterocomplexes in the CA2 and CA3 regions of the dorsal hippocampus, which was linked to the development of significant antidepressant-like effects in the FST. These results give support to the view that 5-HT1A agonists alone can produce antidepressant-like effects involving the CA2 and CA3 areas of the hippocampus (Savitz et al., 2009). A non-significant increase was found after i.c.v. FGF2 alone treatment in these CA regions and in line with these results FGF2 alone failed to produce any significant effects in the FST. Thus, it seems likely that alterations develop in the receptor agonist regulation of the FGFR1-5-HT1A heterocomplexes in the CA2 and CA3 regions in the FSL rat that blocks the recruitment of these complexes upon combined i.c.v. FGF2 and 8-OH-DPAT treatment. It is associated with the lack of development of antidepressant-like effects in the FST upon this combined treatment. Instead, in the FSL rat the 5-HT1A agonist treatment alone should be preferred in view of the antidepressant-like effects observed in the FST linked to a significant recruitment of FGFR1-5-HT1A heterocomplexes in the CA2 and CA3 areas. One reason for these differential actions of the treatments in the FSL and control rat may be that the composition and stoichiometry of these heteroreceptor complexes is different in the two strains. This will likely lead to alterations in their allosteric receptor-receptor interactions which can produce changes in their pharmacology. It should be noted that the FGFR1-5-HT1A heterocomplexes in the CA1 region do not seem to be involved in the antidepressant-like effects observed since they do not change their densities with the agonist treatments performed neither in the FSL nor in the SD rat.

Significant changes were observed in the density of FGFR1-5-HT1A heterocomplexes in the dorsal raphe of the SD rat but not the FSL rat. This should also be considered when discussing the mechanisms for the antidepressant-like effects seen in the FST with combined i.c.v. treatment of FGF2 and the 5-HT1A agonist in the SD rat but not in the FSL rat. In this case, the heteroreceptor complexes contain the 5-HT1A autoreceptor and not the postjunctional 5-HT1A receptor. The activation of the 5-HT1A autoreceptor protomer in the dorsal raphe by the 5-HT1A agonist with or without FGF2 given i.c.v. produced significant increases in the density of PLA clusters in the midbrain raphe 5-HT nerve cells. It is unclear, however, how they relate to the antidepressant-like effects seen in the FST, since in the SD rat they were only observed upon the combined i.c.v treatment.

It may be that in the dorsal raphe cells an increase in the density of the FGFR1-5-HT1A heterocomplexes is not sufficient to elicit the antidepressant-like actions. The combined treatment may be necessary in order to produce through FGFR1 activation a significant uncoupling of the 5-HT1A autoreceptor to the GIRK channel. Indeed, electrophysiological studies *in vivo* and in midbrain slices indicate that stimulation of 5-HT receptors hyperpolarizes dorsal raphe 5-HT neurons, by activating an inwardly rectifying K^+^ conductance mediated by GIRK channels (Aghajanian and Vandermaelen, 1982; Aghajanian and Lakoski, 1984; Williams et al., 1988; Penington et al., 1993a,b; Bayliss et al., 1997). In keeping with these findings, it has been shown that GIRK1, GIRK2 and GIRK3 are expressed in dorsal raphe 5-HT neurons (Karschin et al., 1996; Fairchild et al., 2003; Saenz del Burgo et al., 2008). GIRK2 deletion in dorsal raphe neurons increases the basal firing rate, while GIRK2 mutant mice show a depression-resistant phenotype (Llamosas et al., 2015). Therefore, an increase in the firing of the ascending 5-HT neurons and restoration of 5-HT neurotransmission in the tel-and diencephalon and return of antidepressant like effects may occur by uncoupling 5-HT1A autoreceptor from the GIRK channel in dorsal raphe. To confirm this hypothesis, new electrophysiological experiments in dorsal raphe 5-HT neurons will be needed for evaluating the combined effects of 5-HT1A and FGFR agonists on GIRK currents. In addition, only combined treatment increases the PLA positive FGFR1-5-HT1A heterocomplexes in the CA2 area, which may be essential for antidepressant-like activity observed in the SD rat.

Future work will require the use of interface interfering peptides (Borroto-Escuela et al., 2012) to finally determine the role of FGFR1-5-HT1A heterocomplexes in the FSL genetic model of depression. Moreover, further experiments will be needed to perform electrophysiological analyses of these heterocomplexes in the dorsal raphe and in the hippocampus of the FSL model of depression.

The combined results obtained are compatible with the view that in a genetic model of depression using the FSL rat strain, alterations may develop in the agonist regulation of the density of FGFR1-5-HT1A heteroreceptor complexes located in the dorsal raphe and in the CA2-CA3 areas of the dorsal hippocampus. It is proposed that the lack of antidepressant-like effects seen in the FSL rats upon combined i.c.v. FGF2 and 5-HT1A receptor agonist treatment is related mainly to the failure to increase the density of these heteroreceptor complexes in the dorsal raphe. It may reflect the development of altered allosteric receptor-receptor interactions failing to uncouple the 5-HT1A autorececptors from the GIRK channels. Instead antidepressant-like effects were observed in FSL rats after treatment with 5-HT1A receptor agonist alone. This action may instead be linked to the increases observed after this treatment in the heteroreceptor complexes in the CA2-3 areas of the hippocampus. Due to possible changes in the composition of these complexes vs. those of SD rats leading to altered receptor-receptor interactions, these complexes may respond to the 5-HT1A agonist treatment in a different way from controls and assist in mediating the antidepressant-like effects observed through alterations in the firing of the pyramidal nerve cells.

## Author Contributions

We confirm and declare that all authors meet the criteria for authorship according to the ICMJE, including approval of the final manuscript, and they take public responsibility for the work and have full confidence in the accuracy and integrity of the work of other group authors. They have substantially contributed to the conception or design of the work. Also they have participated in the acquisition, analysis and interpretation of data for the current review version. They have also helped revising it critically for important intellectual content; and final approval of the version to be published. In addition, they have contributed in this last version of the manuscript in writing assistance, technical editing and language editing. DOB-E, CMD, XL, DS, MDP, DL, YA-T, EB, IS, MN, RC, YO, MP, PA, ML and KF designed methods and experiments, carried out the laboratory experiments, analyzed the data and interpreted the results. DOB-E, XL, EB, MN and KF co-designed and co-worked on the radioligand binding experiments. DOB-E, DS, MDP, YA-T, MN, YO, MP, ML and KF co-designed and co-worked on the immunolabeling experiments. DOB-E, CMD, DS, MN, MDP, IS, YO, MP, RC, PA, ML and KF co-designed, discussed analyses, interpretation and presentation of all immunohistochemistry and *in situ* Proximity ligation assay experiments. DOB-E, DS, MN, MDP and KF designed and directed its implementation of the quality assurance and control of each used antibody. DS, DL, YA-T, RC, MDP and PA designed, analyzed the data and interpreted all the electrophysiological studies. CMD, IS and ML designed, analyzed the data and interpreted all the behavioral studies. KF, PA, ML and DOB-E wrote the article. All authors have contributed to, seen and approved the manuscript.

## Conflict of Interest Statement

The authors declare that the research was conducted in the absence of any commercial or financial relationships that could be construed as a potential conflict of interest.
