# Supplementary material for: Disturbances in the FGFR1-5-HT1A Heteroreceptor Complexes in the Raphe-Hippocampal 5-HT System Develop in a Genetic Rat Model of Depression
Source: Front Cell Neurosci. 2017 Oct 10;11:309. doi: 10.3389/fncel.2017.00309 (PMC5641403; doi:10.3389/fncel.2017.00309)
Supplement: Supplementary file 1 [file Data_Sheet_1.pdf]

Supplementary Material

Supplementary Figure 1. For ICV drug delivery animals were implanted with a guide cannula. Rats were anesthetized with isoflurane through a breathing mask. For some FSL rats, pentobarbitate was given to achieve full anaestesia. Guide cannulas were implanted at the following coordinates relative to bregma and the dura surface: mediolateral: - 1.2 mm; anteroposterior: –1.0 mm; dorsoventral: – 3.7 mm; at a 0° angle from the vertical axis in the coronal plane (Paxinos and Watson, 1998). Animals were allowed to recover 6-7 days after surgery before experimental testing. Drugs were delivered 24 hours before the testing day of the forced swim test (i.e. right after the training session) and then again 24 hours before sacrifice and collection of the brain (i.e. right after the testing session).

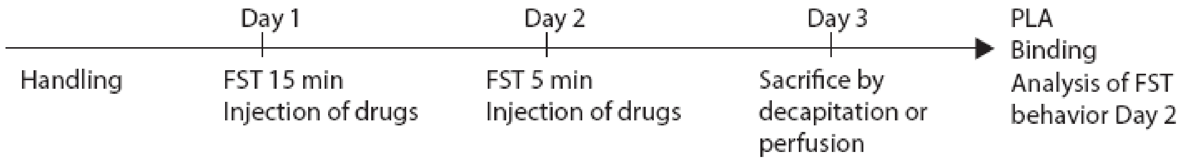

Supplementary Figure 2. The current trace shown in Figure 1A is relative to one recorded cell, showing the variation of holding current when 5-HT1A agonist (8-OH-DPAT) was applied. The holding current using an averaged value per each minute of recordings is illustrated below.

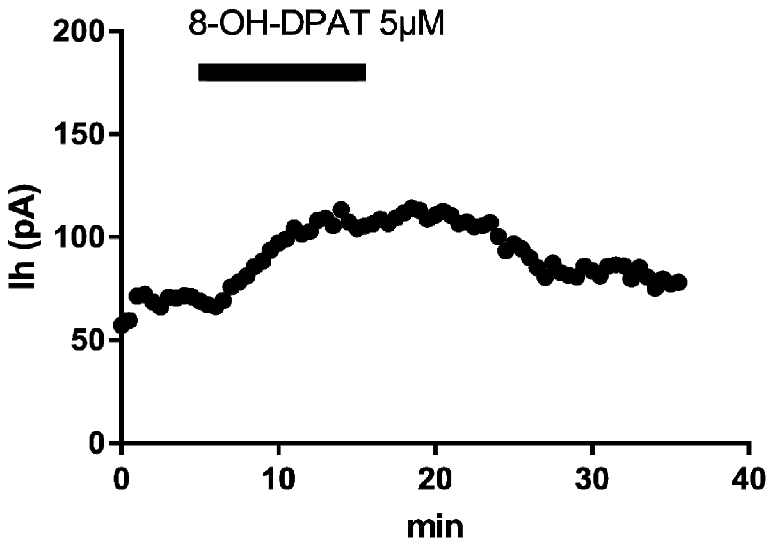

Supplementary Figure 3. Illustration of the decrease of the input resistance produced by the 5-HT1A agonist.

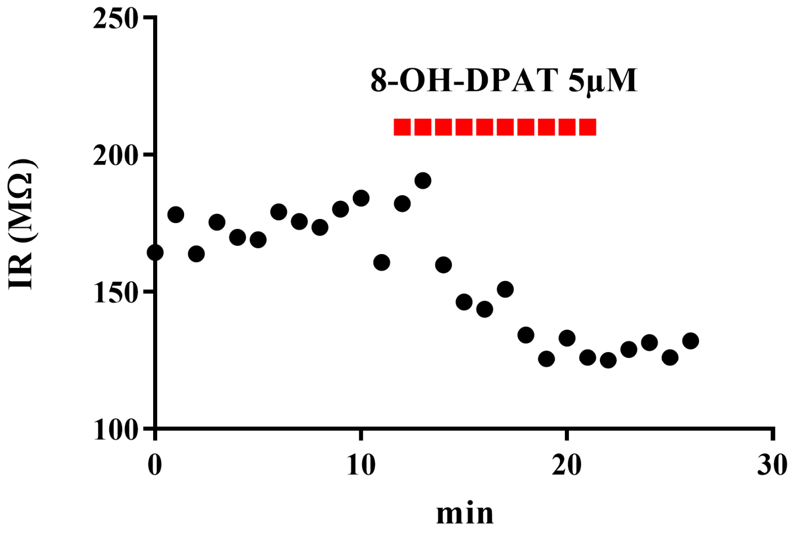

Supplementary Figure 4. Illustration of the linear relationship (red dashed line) between the mean values of delta input resistance (ΔIR) plotted against the mean value of delta holding current (ΔIh) recorded in the experimental groups, which displays how an increase of ΔIh, due to GIRK channel opening, corresponds a decrease of ΔIR.

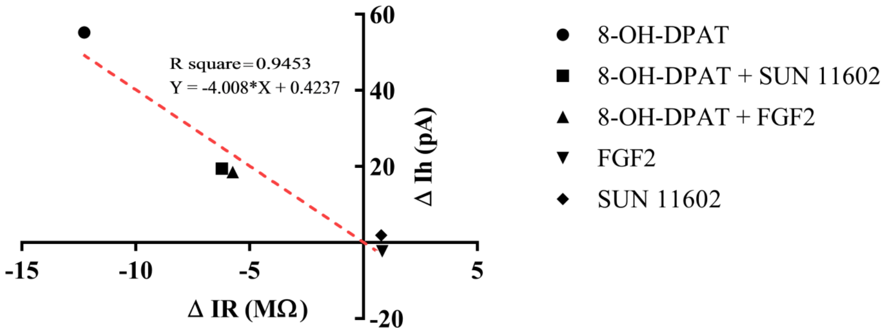

95% Confidence Intervals	
Slope	-5.78 to -2.237
Y-intercept	-11.42 to 12.26
X-intercept	-2.284 to 4.742
Goodness of Fit	
R square	0.9453
Sy.x	6.126
Equation	Y = -4.008*X + 0.4237
